# Preparation and Characterization of Zirconia-Coated Nanodiamonds as a Pt Catalyst Support for Methanol Electro-Oxidation

**DOI:** 10.3390/nano6120234

**Published:** 2016-12-02

**Authors:** Jing Lu, Jianbing Zang, Yanhui Wang, Yongchao Xu, Xipeng Xu

**Affiliations:** 1 Institute of Manufacturing Engineering, Huaqiao University, Xiamen 361021, China; diamond_wangyanhui@163.com (Y.W.); beyondmyselfxyc@163.com (Y.X.); xpxu@hqu.edu.cn (X.X.); 2 State Key Laboratory of Metastable Materials Science & Technology, Yanshan University, Qinhuangdao 066004, China

**Keywords:** nanodiamond, ZrO_2_ shell, deposition, Pt nanoparticles, oxidation

## Abstract

Zirconia-coated nanodiamond (ZrO_2_/ND) electrode material was successfully prepared by one-step isothermal hydrolyzing from ND-dispersed ZrOCl_2_·8H_2_O aqueous solution. High-resolution transmission electron microscopy reveals that a highly conformal and uniform ZrO_2_ shell was deposited on NDs by this simple method. The coating obtained at 90 °C without further calcination was mainly composed of monoclinic nanocrystalline ZrO_2_ rather than common amorphous Zr(OH)_4_ clusters. The ZrO_2_/NDs and pristine ND powder were decorated with platinum (Pt) nanoparticles by electrodeposition from 5 mM chloroplatinic acid solution. The electrochemical studies indicate that Pt/ZrO_2_/ND catalysts have higher electrocatalytic activity and better stability for methanol oxidation than Pt/ND catalysts in acid.

## 1. Introduction

Direct methanol fuel cells (DMFCs) have attracted more and more attention for their reduced weight, high energy efficiency, low pollutant emissions, and easy transportation and storage as future power sources [1,2,3]. However, there are still two key problems inhibiting their commercial applications: the cost of the precious metals employed and the slow reaction kinetics caused by catalyst poisoning [4,5,6]. So it is necessary to prepare some more effective catalyst supports with sufficiently high activity and CO tolerance for methanol electro-oxidation.

Nanostructured carbon materials [7,8,9,10,11], including carbon nanotubes, mesoporous carbon and graphene, have already been commonly used as supports to minimize the use of precious metals. However, sp^2^-bonded carbon is susceptible to corrosion and microstructural degradation during anodic polarization, leading to a loss of activity or even electrode failure [12]. Alternatively, sp^3^-bonded nanodiamonds (NDs) possessing unparalleled stability is a more promising candidate for an electrode material because of its wide potential window together with its low background current [13,14,15,16]. In our previous work, Pt/ND catalysts with proper Pt mass loading exhibited a good electrocatalytic effect on methanol oxidation [17]. Nevertheless, the relatively low electrochemical activity and poisoning CO are still the main obstacles that need to be conquered.

Recently, many studies have been reported to prepare catalysts with higher activity toward methanol electro-oxidation and they have found that the addition of oxides to metallic Pt is efficient to increase CO tolerance through synergetic interaction [18,19,20,21,22]. ZrO_2_ as a material which has a high melting point, outstanding chemical stability, and excellent mechanical properties is also a promoter for the preferential oxidation of CO [23,24,25], and it can be used in both alkaline and acid solutions. Since nearly all these oxides involve heat treatment at high temperatures in aerobic atmosphere, they may cause thermal damage to carbon supports.

In this paper, we synthesized ZrO_2_-coated NDs (ZrO_2_/NDs) by isothermal hydrolyzing [26] of ND-dispersed ZrOCl_2_·8H_2_O aqueous solution at 90 °C without calcination. The morphology, structure and composition of the ZrO_2_/NDs were characterized by high-resolution transmission electron microscopy (HRTEM), selected area electron diffraction (SAED) and energy dispersive spectroscopy (EDS). The electrocatalytic effects of Pt catalysts supported on ZrO_2_/NDs and NDs for methanol electro-oxidation were comparatively studied by electrochemical experiments.

## 2. Experimental

### 2.1. Preparation of ZrO_2_/ND

The used mechanical milling ND particles are commercial products (Guangzhou Advapowder Nano-Technology Company Ltd., Guangzhou, China) with an average primary size of 100 nm. Solid ZrOCl_2_·8H_2_O employed as the precursor for the synthesis of ZrO_2_ was first dissolved in 100 mL distilled water to produce 0.2 mol/L solution under magnetically stirring. ND powder of 100 mg without any pretreatment was then added into the aqueous solution. After 30 min ultrasonic vibration, a stable suspension with diamond particles homogeneously dispersed was obtained. By reflux condensing in a thermostatic water bath at the temperature of 90 °C for 96 h, the grey suspension turned to hoar, revealing the deposition of the ZrO_2_ coating. The resultant samples were filtrated and washed with deionized water and ethanol repeatedly, and then dried 24 h at room temperature in air.

### 2.2. Characterization of ZrO_2_/ND

The structure and morphology observation of ZrO_2_/ND was performed on a JEM-2010 HRTEM (JEOL Ltd, Tokyo, Japan) at 200 kV accelerating voltage and the images were recorded digitally with a charge-coupled device camera. The chemical component of the composites was also examined by an EDS (Oxford Instruments plc, Oxon, UK) equipped on the TEM (JEOL Ltd, Tokyo, Japan). The specimens were prepared by dispersing the nanopowders in ethanol with ultrasonic treatment, followed by dropping on the holey carbon-coated copper grids.

### 2.3. Electrodeposition of Pt Catalysts

ZrO_2_/ND (or ND) powder was immersed in 10 mL ethanol by ultrasonication for 30 min to achieve a 1.0 mg/mL suspension. One drop of the suspension was cast on a glassy carbon (GC) electrode (2 cm in diameter) and dried at 80 °C to prepare ZrO_2_/ND (or ND) modified GC electrode. Pt nanoparticles were electrodeposited on the ZrO_2_/ND (or ND) electrode by a potentiostatic method from 5 mM H_2_PtCl_6_ in 0.5 M H_2_SO_4_ solution. The deposition potential was −0.2 V (vs. Ag/AgCl). After 1200 s electrodeposition, the electrode was washed in deionized water. The morphology of the electrode was observed by an S-4800 field emission scanning electron microscope (FESEM, S-4800, Hitachi Ltd, Tokyo, Japan).

### 2.4. Electrochemical Measurement

All electrochemical measurements were performed on a CHI660A electrochemical workstation. A conventional three-electrode system was used, consisting of a Pt/ZrO_2_/ND (Pt/ND) electrode as the working electrode, a platinum coil auxiliary electrode, and an Ag/AgCl electrode as the reference electrode. The experiments were conducted at 25 °C. All solutions were prepared using analytical grade reagents in deionized water and were saturated with nitrogen gas before use. Comparative electrocatalytic activities of Pt/ZrO_2_/ND and Pt/ND electrocatalysts for methanol electro-oxidation were measured by cyclic voltammetry (CV) in 0.5 M CH_3_OH + 0.5 M H_2_SO_4_ solution.

## 3. Results and Discussion

**Figure 1:**
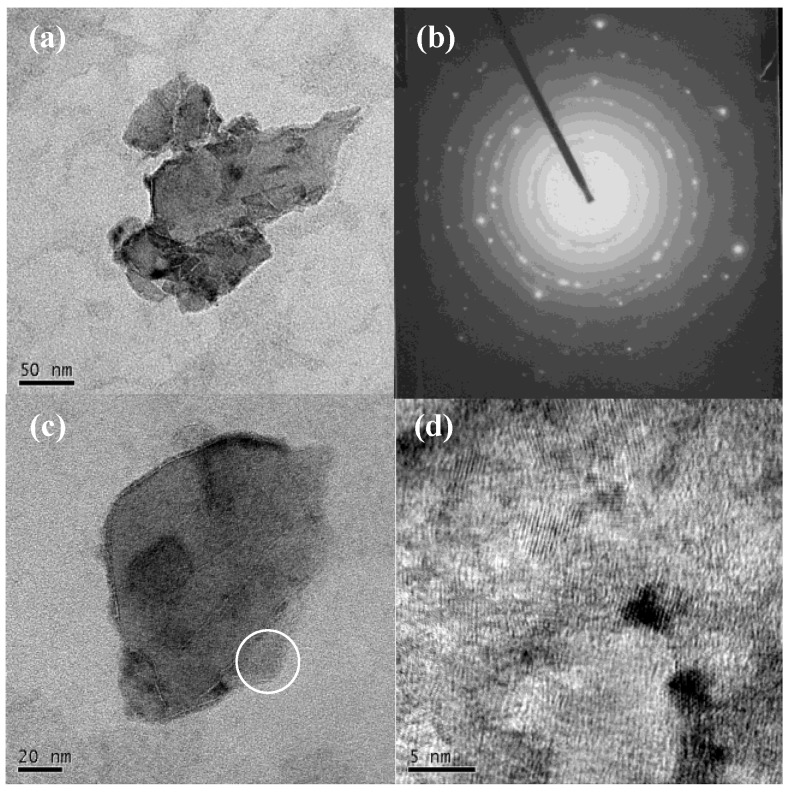
Transmission electron microscopy (TEM) (**a**,**c**); selected area electron diffraction (SAED) (**b**); and high-resolution TEM (HRTEM) (**d**) images of zirconia-coated nanodiamond (ZrO_2_/ND).

Figure 1 shows the TEM, SAED and HRTEM images of ZrO_2_-coated NDs. Nearly all the diamond particles are fully and homogeneously covered with ZrO_2_ coating by isothermal hydrolysis for 96 h. Moreover, there are no flocculent ZrO_2_ aggregates among the ND particles. The SAED result demonstrates that the ZrO_2_ coating on NDs is mainly composed of monoclinic nanocrystalline zirconia rather than hydrous or amorphous zirconia.

In isothermal hydrolyzing, the slow and soft reaction promotes the adsorption of the species on the ND surface and the subsequent condensation of the adsorbed layer. Therefore, the nucleation and growth of the species tend to occur on the ND surface rather than in the suspension. Unlike the two mixed phases obtained from the traditional chemical precipitation process, a core-shell structural nanocomposite could be achieved with this synthetic method.

The magnified image of a single ZrO_2_/ND particle in Figure 1c suggests that the coated ZrO_2_ layer has perfect conformability with the ND substrate and the thickness of the coating is approximately 5 nm. Nevertheless, the coating in the circle-marked area is much thicker, probably because the defects on the ND surface accelerate the formation of the zirconia nuclei. The corresponding HRTEM image of this selected part illustrates that the lattice spacing of ZrO_2_ is 0.28 nm, which is consistent with the (111) of m-ZrO_2_.

The EDS spectrum taken from the same ZrO_2_/ND indicates the coexistence of elements C, O and Zr in the sample. The C comes from the diamond core, while the Zr and O are from the zirconia shell. Since the coating layer is ultrathin, the amount of zirconia is quite small. The Zr/O atomic ratio is 1.24/2.41 (≈1/1.94), nearly equal to the Zr/O atomic ratio in ZrO_2_. This further confirms that the coating deposited by isothermal hydrolysis is not common hydrolyzing acquired amorphous Zr(OH)_4_ but ZrO_2_.

**Figure 2:**
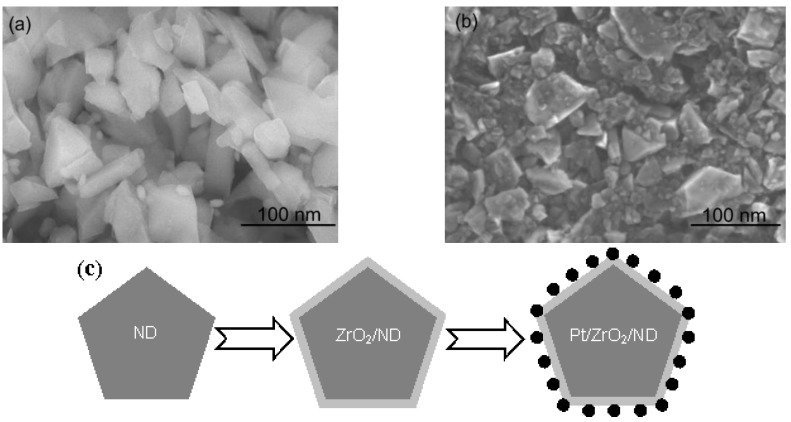
Field emission scanning electron microscope (FESEM) images of ZrO_2_/ND (**a**); Pt/ZrO_2_/ND (**b**); and the formation process of Pt/ZrO_2_/ND (**c**).

The FESEM images of the ZrO_2_/ND and Pt/ZrO_2_/ND electrodes are separately shown in Figure 2a,b. They reveal that the pristine ND powders are uniformly wrapped by zirconia coating. After electrodeposition, the Pt catalysts are well dispersed on the surface and the particle size is around 5–8 nm. Figure 2c schematically represents the formation process of the Pt/ZrO_2_/ND electrode material. After coating the nanodiamond core with a zirconia shell, Pt catalyst particles were electrodeposited onto the surface. Compared to pure Pt catalysts, the obtained Pt nanoparticles on the supports possess a more effective surface and would not aggregate together, especially when the ZrO_2_/ND support material is a nanometer-scale powder.

**Figure 3:**
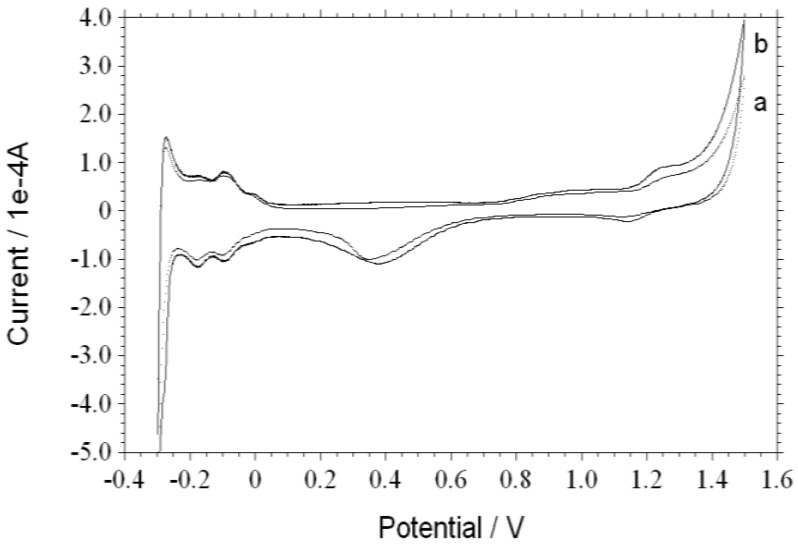
Cyclic voltammetry (CV) curves of Pt/ND (**a**) and Pt/ZrO_2_/ND (**b**) electrodes in 0.5 mol/L H_2_SO_4_.

Figure 3 shows the recorded CV curves of the Pt/ND and Pt/ZrO_2_/ND electrodes in 0.5 M H_2_SO_4_ solution after 1200 s eletrodeposition. The scan rate is 0.1 V/s in the potential range of −0.3 to 1.5 V. They both display the well-known characteristic peaks of the bulk Pt electrodes. The Pt oxide formation is in the +0.8 to +0.9 V region and the reduction of Pt oxide is from +0.3 to +0.4 V, suggesting the successful deposition of Pt particles by this potentiostatic method. Otherwise, the area of hydrogen adsorption at 0 to −0.3 V for Pt/ZrO_2_/NDs is larger than that for Pt/NDs under the same deposition conditions. Since the active specific surface area of Pt particles can be signified by the integrated charge in the hydrogen adsorption region of the CV, the Pt/ZrO_2_/NDs are expected to show better catalytic activity.

**Figure 4:**
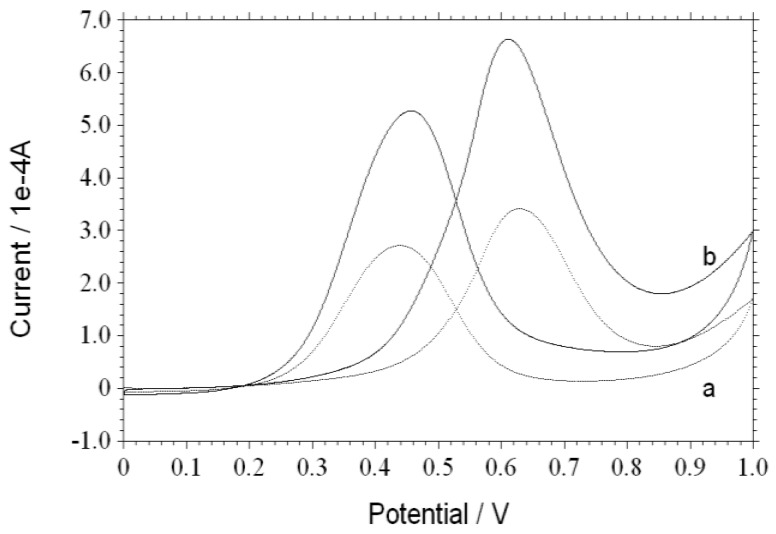
CV curves of Pt/ND (**a**) and Pt/ZrO_2_/ND (**b**) electrodes in 0.5 mol/L CH_3_OH + 0.5 mol/L H_2_SO_4_.

**Figure 5:**
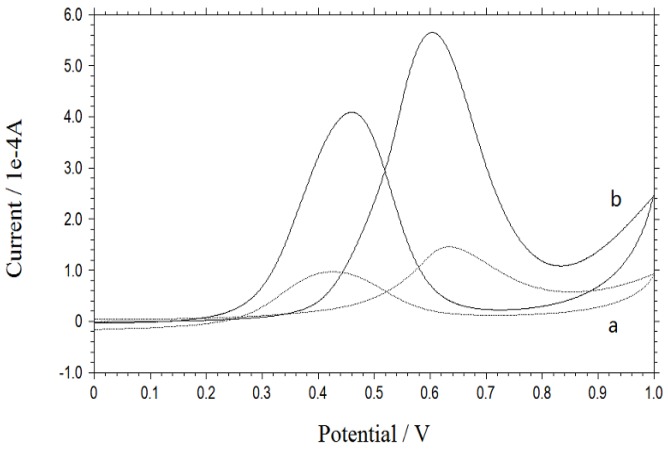
CV curves of Pt/ND (**a**) and Pt/ZrO_2_/ND (**b**) electrodes in 0.5 mol/L CH_3_OH + 0.5 mol/L H_2_SO_4_ after 500 cycles.

The typical CV curves for methanol electro-oxidation on Pt/NDs and Pt/ZrO_2_/NDs in 0.5 M CH_3_OH + 0.5 M H_2_SO_4_ aqueous solution are shown in Figure 4. The scan rate is 0.05 V/s in the potential range of 0 to 1.0 V. For Pt/NDs, methanol oxidation begins at approximately 0.46 V and reaches a peak current at about 0.63 V. On the reverse sweep, re-oxidation of methanol begins at approximately 0.60 V and reaches its current peak at around 0.44 V. Comparatively, the peak potentials are respectively 0.61 and 0.45 V for Pt/ZrO_2_/NDs. Although the electrochemical behavior of Pt/ZrO_2_/NDs is similar to that of Pt/NDs, the peak current of the former one is significantly higher. The peak current for Pt/ZrO_2_/NDs is 5.00 × 10^−4^ A, while the peak current for Pt/NDs is 2.55 × 10^−4^ A. After 500 cycles shown in Figure 5, the peak current for Pt/ZrO_2_/NDs is 4.79 × 10^−4^ A, while the peak current for Pt/NDs is reduced to 0.99 × 10^−4^ A. The decreasing rate of the Pt/ZrO_2_/NDs was much lower, indicating the highest stability of the Pt/ZrO_2_/ND electrode. All these facts prove that the ZrO_2_ coating contributes to the higher catalytic activity and better CO tolerance for methanol oxidation.

Compared to TiO_2_/NDs or other carbon substrates in our previous work [27,28], the ZrO_2_ layer on the ND surface improved the interaction of the support with Pt metal, and the conformal structure of the coating acquired without heat treatment avoided not only the agglomeration of Pt NPs but also the thermal damage to NDs. Therefore, the high stability of Pt/ZrO_2_/NDs can be ascribed to the anchoring effect and the charge transfer provided by ZrO_2_ coating.

## 4. Conclusions

A zirconia shell has been deposited on the ND core in ZrOCl_2_·8H_2_O aqueous solution. By this one-step isothermal hydrolyzing method, a uniform monoclinic nanocrystalline ZrO_2_ coating has been obtained rather than common amorphous Zr(OH)_4_ clusters. The results of CV show that the Pt/ZrO_2_/ND catalyst has a higher peak current and lower peak potential than Pt/NDs for methanol electro-oxidation. The addition of the ZrO_2_ interlayer significantly improves the catalytic activity and CO tolerance of the electrode. Considering its high electrochemical and thermal stability, ZrO_2_/ND powder may be good a candidate as a catalyst support.
